# Elevated Neopterin Levels Predict Fatal Outcome in SARS-CoV-2-Infected Patients

**DOI:** 10.3389/fcimb.2021.709893

**Published:** 2021-08-23

**Authors:** Manon Chauvin, Martin Larsen, Bibiana Quirant, Paul Quentric, Karim Dorgham, Luca Royer, Hélène Vallet, Amelie Guihot, Béhazine Combadière, Christophe Combadière, Jaume Barallat, Julien Mayaux, Charles-Edouard Luyt, Alexis Mathian, Zahir Amoura, Jacques Boddaert, Fernando Armestar, Guy Gorochov, Eva Martinez-Caceres, Delphine Sauce

**Affiliations:** ^1^Sorbonne Université, Inserm, Centre d’Immunologie et des Maladies Infectieuses, Cimi-Paris, Paris, France; ^2^Division of Immunology, Germans Trias i Pujol University Hospital and Research Institute, Badalona, Spain; ^3^Department of Cellular Biology, Physiology and Immunology, Universitat Autònoma de Barcelona, Bellaterra (Cerdanyola del Vallès), Spain; ^4^Assistance Publique-Hôpitaux de Paris (AP-HP), Hôpital Saint-Antoine, Unité de Gériatrie Aigue, Paris, France; ^5^Assistance Publique-Hôpitaux de Paris (AP-HP), Groupement Hospitalier Pitié-Salpêtrière, Département d’Immunologie, Paris, France; ^6^Biochemistry Department, Germans Trias i Pujol University Hospital, Badalona, Spain; ^7^Assistance Publique-Hôpitaux de Paris (AP-HP), Groupement Hospitalier Pitié-Salpêtrière, Service de Médecine Intensive–Réanimation et Pneumologie, Paris, France; ^8^Service de Médecine Intensive Réanimation, Institut de Cardiologie, Assistance Publique–Hôpitaux de Paris (APHP), Sorbonne-Université, Service de Médecine Intensive–Réanimation et Pneumologie, Paris, France; ^9^Service de Médecine Interne 2, Institut E3M, Assistance Publique Hôpitaux de Paris (AP-HP), Hôpital Pitié-Salpêtrière, Paris, France; ^10^Assistance Publique–Hôpitaux de Paris (APHP), Sorbonne-Université, Hôpital Pitié–Salpêtrière, Département de Gériatrie, Paris, France; ^11^Critical Care Department, Germans Trias i Pujol University Hospital, Badalona, Spain; ^12^Department of Medicine Universitat Autònoma de Barcelona, Bellaterra (Cerdanyola del Vallès), Spain

**Keywords:** SARS-CoV-2, biomarker, neopterin, clinical outcome, death

## Abstract

**Highlights:**

Innate immune activation during Covid-19 infection is associated with pernicious clinical outcome.

**Background:**

Coronavirus disease 2019 (Covid-19) is a worldwide threat that has already caused more than 3 000 000 deaths. It is characterized by different patterns of disease evolution depending on host factors among which old-age and pre-existing comorbidities play a detrimental role. Previous coronavirus epidemics, notably SARS-CoV, were associated with increased serum neopterin levels, which can be interpreted as a sign of acute innate immunity in response to viral infection. Here we hypothesize that neopterin may serve as a biomarker of SARS-CoV-2 viral infection and Covid-19 disease severity.

**Methods:**

We measured neopterin blood levels by ELISA. Seric concentration was quantified from 256 healthy donors and 374 Covid-19 patients at hospital admission. Enrolled Covid-19 patients were all symptomatic and displayed a large spectrum of comorbidities. Patients were followed until disease resolution or death.

**Results:**

Severe and critically ill SARS-CoV-2 infected patients were characterized by a profound exacerbation of immune activation characterized by elevated neopterin blood levels. Systemic neopterin levels above 19nM stratified healthy individuals from Covid-19 patients with 87% specificity and 100% sensitivity. Moreover, systemic neopterin levels above 53nM differentiated non-survivors from survivors with 64% specificity and 100% sensitivity.

**Conclusion:**

We propose that neopterin concentration measured at arrival to hospital is a hallmark of severe Covid-19 and identifies a high-risk population of pernicious clinical outcome with a need for special medical care.

## Introduction

The severe acute respiratory syndrome coronavirus 2 (SARS-CoV-2) disease (Covid-19) has rapidly spread from China to the rest of the world. The resulting pandemic has challenged societies and their health care systems and requested unprecedented political, medical and scientific actions. The long-term societal and economic consequences of the pandemic per country start to emerge.

SARS-CoV-2 is an RNA virus of the *Coronaviridae* family, which is closely related with SARS (SARS-CoV) and Middle East respiratory syndrome-related coronavirus (MERS-CoV). These viruses are airborne and they all infects epithelium (primarily lung epithelium) through a high affinity interaction between the viral spike protein and cell surface expressed angiotensin converting enzyme 2 (ACE2). Viral entry is finally facilitated through proteolytic cleavage of ACE2 *via* the host type II transmembrane serine metalloprotease TMPRSS2 (Heurich et al., 2014) which equally cleaves the SARS-CoV-2 spike protein (Hoffmann et al., 2020).

SARS-CoV-2 infection leads to disease courses varying from none or mild symptoms for the majority of infected individuals to severe or critical disease. Mild cases most often recover without medical intervention. Among the minority with severe symptoms a non-negligible fraction, particularly among older and comorbid individuals, has fatal outcome (Verity et al., 2020).

SARS-CoV-2 infected individuals will undergo a non-symptomatic incubation period lasting on average 6.4 days (Lescure et al., 2020). During this incubation period the virus will replicate and the exposed individual will transition from non-infectious to infectious. Infected individuals generally suffer a mild disease course associated with viremia in the respiratory tracts for 8-10 days. These patients will either recover or transition towards a more severe disease phase characterized by prolonged viremia (Lescure et al., 2020) and a pronounced pro-inflammatory signature (Chen et al., 2020). The disability to control the cytokine storm ravaging during the severe phase of the disease is believed to be the primary cause of fatality.

Despite efforts to develop therapeutic approaches the world has largely faced the first 9-12 months of pandemic with no efficient prophylactic or therapeutic medical intervention available. Indeed, the primary purpose of the health care system has been to provide life support to ensure the time for the patient to naturally clear the virus. Life support consists primarily of providing oxygen and sometimes mechanical ventilation.

During a pandemic, it is of outmost importance not to surpass the capacity of the health care system for a given country. This is accommodated at two levels; the hospital level and the political level. At the hospital level medical expertise should ensure that hospital resources are distributed effectively and according to patient needs. At the political level one can install temporary reinforced socioeconomic regulations to dampen the propagation of disease through isolation of infected individuals or even confinement of entire populations. The latter strategy has been adopted by most developed countries and serves to extend the period of the pandemic to reduce the number of infected individuals during the peak of the pandemic (Sheikh et al., 2020; Zanin et al., 2020). Meanwhile, overwhelmed hospital wards require tools for patients’ severity stratification.

Stratification of Covid-19 patients on the basis of cellular biomarkers is an unmet need in patient care. The primary objective of this study is to characterize the relationship between neopterin blood levels at hospital admission and clinically assessed severe subsequent evolution. Indeed, coronavirus infections in the past have been characterized by the onset of a cytokine storm. The hallmark of a cytokine storm is an abnormal regulation and uncoordinated release of several pro-inflammatory cytokines at inappropriate time intervals during infection, such as interleukin (IL)-6, IL-1β, Tumor necrosis factor (TNF-α) and type-I interferons (IFNs) (Huang et al., 2005; Hadjadj et al., 2020; Dorgham et al., 2021; Mahat et al., 2021) resulting in an uncontrolled inflammatory response, acute respiratory distress syndrome, respiratory failure, shock, organ failure, and death (Huang et al., 2005; Batisse et al., 2020; Chen et al., 2020; Mortaz et al., 2020; Remy et al., 2020; Wang et al., 2020). It is therefore reasonable to postulate that the inflammatory response measured both at cellular and molecular levels would represent a main prognostic signature for the disease.

Neopterin (1’, 2’, 3’-D-erythro-trihydroxypropylpterin) belongs to the family of pteridines and is involved in a variety of oxidation-reduction reactions in the body. Neopterin is derived *in vivo* from guanosine triphosphate (GTP). The reaction is catalyzed by the enzyme GTP-cyclohydrolase-I (GCH I) in activated monocytes, macrophages, dendritic cells, and endothelial cells upon stimulation mainly by IFN-γ (Fuchs et al., 1993; Murr et al., 2002). Its release by macrophages in response to cytokines released by T lymphocytes and natural killer cells make neopterin an indicator of activation of cell-mediated immunity, which is tightly associated with hyper-inflammation. Therefore, the measurement of neopterin reflects an elevated and likely sustained immune activation taking place during viral assault and cytokine storm. Noteworthy, it has been described that neopterin was associated with the clinical progression of patients infected with SARS-CoV and that the plasmatic levels observed during SARS-CoV infection were predictive of the severity of respiratory syndromes independently of viremia (Zheng et al., 2005).

Here we demonstrate that increased serum neopterin levels at hospital admission are predictive of extended symptomatic disease duration and non-survival post SARS-CoV-2 infection. Neopterin therefore constitutes a relevant prognostic biomarker to optimize Covid-19 patient’s management.

## Materials and Methods

### Study Subjects

A total of 610 individuals took part in this case-control design study including 374 participants with diagnosis of Covid-19 according to WHO criteria and positive SARS-CoV-2 RT-PCR testing on a respiratory sample (nasopharyngeal swab or invasive respiratory sample) as well as 256 age and sex-matched healthy controls. In France SARS-CoV-2-infected patients (n=65) was admitted either to the Department of Emergency, Intensive Care Unit (ICU) or to the Internal Medicine ward at Pitié-Salpêtrière Hospital (Paris, France) for symptomatic SARS-CoV-2 viral infection between March 17 and May 11, 2020. Serum separating tube from blood samples were collected at admission (day of arrival to hospital). In Spain, SARS-CoV-2 infected individuals (n=309) were recruited for Germans Trias i Pujol Hospital, Institut Català de la Salut (Barcelona, Spain) after admission in either the Department of Emergency, ICU or to the Pneumology ward between March 15 to April 11, 2020. For each individual, sera were cryopreserved at -80°C until use. The present study consists of SARS-CoV-2 infected patients with known clinical outcome within three months post hospital admission.

### Data Collection

Data collected prospectively included age, sex, previous medical history, date of symptoms onset, and duration of hospitalization. Associated comorbidities were carefully reviewed, with a particular focus on the history of cardiovascular or respiratory events, diabetes and obesity. Demographic and clinical characteristics are detailed in **Table 1**.

**Table 1:** Patients’ characteristics.

COVID-19	Recovery (n = 298)	Deceased (n = 76)	P°ℵ^2^ test****
**Age**			
Mean (SD)	58 (14)	73 (11)	<0.001
**Gender**			
Female	40% (118)	32% (24)	0.25
**Service**			
Hospital	25.2% (75)	27.7 (96)	0.24
Emergency	57% (170)	47.3% (36)	
ICU	17.7% (53)	25% (19)	
**Past Medical History**			
Cardiovascular Disease	7.7%(23)	19.7% (15)	0.004
Hypertension	35.9% (107)	77.6% (59)	<0.001
Diabetes	17.4% (52)	43.4% (33)	<0.001
Obesity	15.7% (47)	13.1% (10)	0.12
Chronic Respiratory Disease	6% (18)	10.5% (8)	0.26
**Disease Severity**			
Length of Stay [Days (SD)]	15 (13)	12 (14)	0.007
Disease Duration [Days (SD)]	23 (14)	17 (13)	0.002
**Neopterin**			
Mean (SD)	44 (24)	101 (48)	<0.001

### Ethical Statement

The study was conducted in accordance to the Declaration of Helsinki. All participants provided informed consent. The study was registered by local ethical committee of Sorbonne-Université/Assistance Publique des Hôpitaux de Paris for standard hospitalized patients (n° CER2020-21) and ICU patients (n° CER2020-31). Similarly, ethical approval was obtained in Spain by local ethical committee (n°PI20-20).

### Inflammation Measurement

As described in (Dorgham et al., 2021), serum cytokine concentrations were determined in the French samples using quantitative kits from Quanterix (Lexington, MA, USA); IFN-α, IFN-γ, IL-1β, IL-6, IL-8, IL-10, IL-17A, IL-18, IL-22, GM-CSF and TNF-α) and IFN-β ELISA (PBL Assay Science, Piscataway, NJ, USA).

Admission levels of neopterin were determined using a commercially available ELISA kit (Tecan) as previously described (Larsen et al., 2017). All samples were centrally processed and analyzed in Cimi-Paris.

### Statistical Analysis

Groups were compared using a parametric t-test: Paired comparisons were conducted with a non-parametric Wilcoxon test. Differences in survival were analyzed with the Kaplan-Meier method, data on surviving or non-surviving patients were censored at three months post arrival to hospital. Survival curves were compared with a log-rank test. A Cox proportional hazard model was used to compute hazard ratios and associated 95% confidence intervals (CI). *P* values < 0.05 were considered statistically significant.

To quantify the accuracy of our predictive model we plotted receiver operating characteristic (ROC) curves, which depicts the specificity and sensitivity of the prediction variable and indicates the overall quality of the prediction model through the area under the ROC curve (AUC).

All statistical analysis was performed with GraphPad prism software and R studio (v1.4). Graphical representations were performed with the ggplot2 package (v3.3) (Wickham, 2016). ROC analysis was conducted with the pROC package (v1.17) (Robin et al., 2011) and survival models were established with the Survival package (v3.2) (Therneau and Grambsch, 2000).

## Results

### Patients Characteristics

Healthy volunteers (recruited from Etablissement Français du Sang and geriatrics department at Pitié-Salpêtrière hospital) participate to this study as control group (n=256). All were free of acute pathologies. They were aged 63 years on average, with 55% of them being males. SARS-CoV-2 infected patients from France (n=65) and from Spain (n=309) admitted to either ICU departments or non-ICU departments were included. SARS-CoV-2 infection was confirmed on nasopharyngeal swab by positive RT-PCR in accordance with WHO interim guidance. Clinical and biological characteristics are shown in **Table 1** and **Supplementary Table S1**. Mean age was 61 years, with 62% of them being males. Hospital admission occurred on average 8.7 days after the onset of symptoms. The comorbidities covered a large range of common medical illnesses: hypertension (44%), type 2 diabetes (23%), obesity (13%), cardiovascular disease (10%) and chronic respiratory disease (7%). 79% of all patients were discharged before the 18^th^ of June 2020, and 20.3% had died.

**Figure 1:**
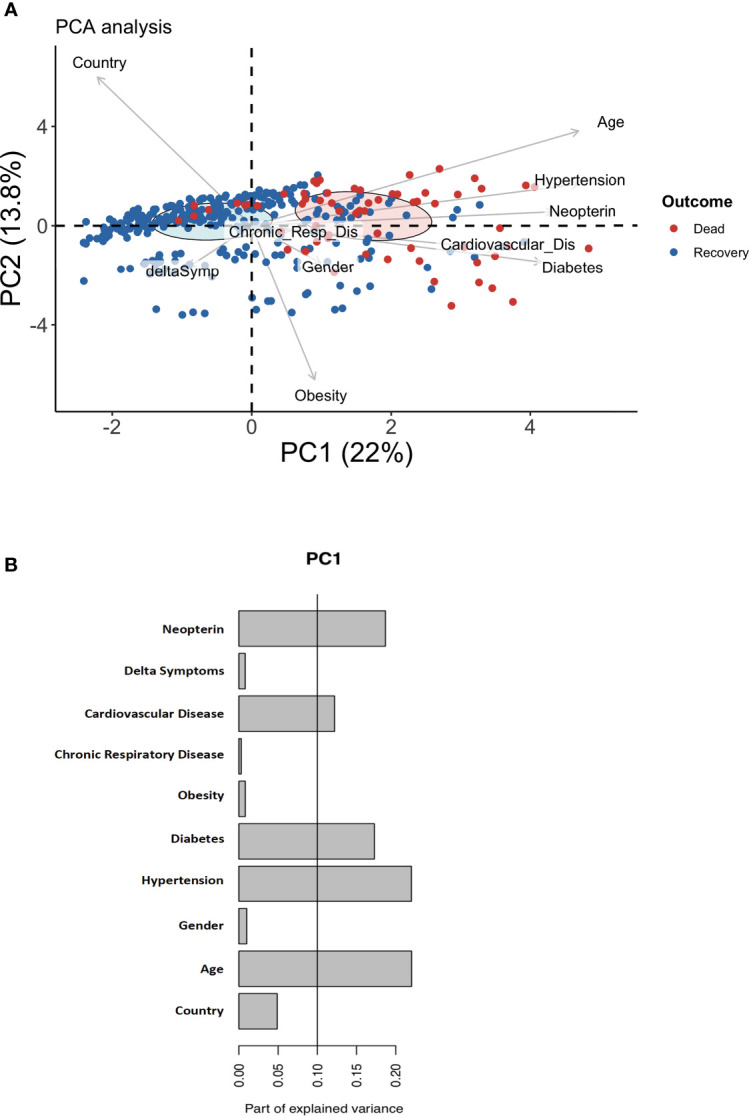
Discriminating parameters to segregate Covid-19 suffering patents from healthy individuals. **(A)** Principal component analysis (PCA) using serum neopterin and clinical variables on hospitalized patients. Red symbols represent deceased patients. Blue symbols represent patients who recovered within one month-follow-up (survivors). **(B)** Histograms depicting the contribution of individual PCA parameters with regards to the variance of principal component 1 (PC1).

To better evaluate the overall clinical profile, we conducted a principal component analysis (PCA) to determine if certain combinations of comorbidities would allow for a stratification of the patients according to fatal clinical outcome. The first principal component allowed to make a clear distinction between Covid-19 patients who recovered from those who died (**Figure 1A**), with elevated levels of neopterin as discriminant parameter (**Figure 1B**). As expected, age, hypertension, diabetes and cardiovascular disease also contribute to the principal component enabling the distinction between patients who recovered and those who died (**Figure 1B**). Of note, sample origin (country) did not explain variance associated with clinical outcome, suggesting that treatment variability between France and Spain did not impact overall survival. When comparing survivors *vs* non-survivors, we noticed that deceased patients were older than survivors (73 *vs* 58 years old, p<0.001) and suffer more of comorbidities (78% *vs* 36% for hypertension, p<0.001; 43% *vs* 17% for diabetes, p<0.001 and 20% *vs* 7.7% for cardiovascular disease, p=0.004; **Table 1**).

### Neopterin as Biomarker of Acute SARS-CoV-2 Infection

**Figure 2:**
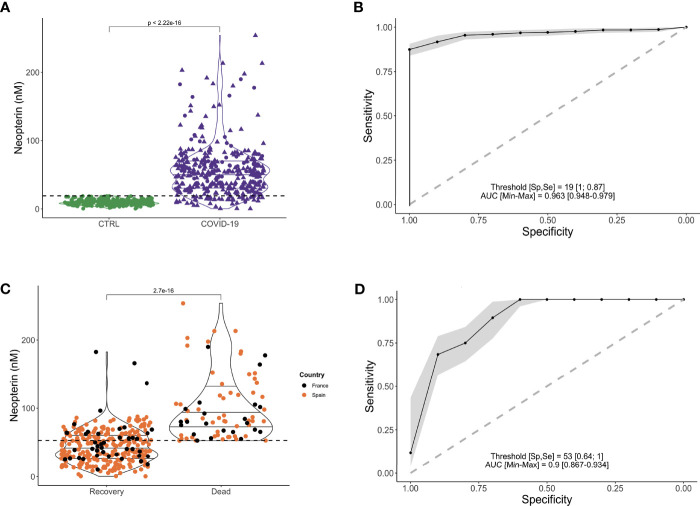
Higher serum level of neopterin is associated with Covid-19 infection and fatal outcome. **(A)** Violin plot representing serum neopterin levels (in nM) stratified according to infectious status (CTRL (uninfected healthy donors, uninfected, in green); Covid-19 (SARS-CoV-2 infected individuals, in purple). Shape of dots represents country of inclusion of infected patients (Circles for France; Triangles for Spain). Dotted line shows the threshold (19nM) enabling the stratification of healthy *vs* infected patients. **(B)** Receiver Operating Characteristics (ROC) curve depicting sensitivity and specificity of prediction model for SARS-CoV-2 infection based on neopterin level. Grey zone represents confidence interval. AUC= 0.963; Specificity = 100%; Sensitivity= 87%; threshold = 19nM. **(C)** Violin plot representing serum neopterin levels (in nM) stratified according to clinical outcome (RECOVERY (survivors) or DEAD (non-survivors). Colors of dots represent country of inclusion of infected patients (Black circles for France; Orange circles for Spain). Dotted line shows the threshold (53nM) enabling the stratification of deceased *vs* survivors. **(D)** Receiver Operating Characteristics (ROC) curve depicting sensitivity and specificity of prediction model for fatal outcome based on neopterin level. Grey zone represents confidence interval. AUC = 0.94; Specificity = 100%; Sensitivity = 64%; threshold = 53nM.

As shown in **Figure 2A**, the concentration of neopterin at admission was statistically significant between the control group of healthy volunteers and the symptomatic Covid-19 patients (9.5nM *vs* 56nM; p=2.10^-16^).

The level of systemic neopterin measured in patients at their arrival to hospital is a strong predictor of infection based on a ROC analysis (**Figure 2B**; AUC=0.963). The predictor has 87% sensitivity and 100% specificity with a cut-off value set at 19 nM.

### Neopterin as Biomarker of Fatal Outcome

Within SARS-CoV-2 infected patients, neopterin was able to stratify for the severity of the disease based on survival of the patients. As shown in **Figure 2C**, the concentration of neopterin at admission was statistically significant between the group of symptomatic Covid-19 patients who died compared to survivors (101nM *vs* 44nM; p=2.7x10^-16^). Multiparametric logistic regression analysis demonstrated that neopterin’s ability to predict pernicious clinical outcome is independent of comorbidities, such as obesity, diabetes, respiratory disease and cardiovascular disease (p<10^-6^). Indeed, despite a significant difference of survival depending on the co-morbidities status, the predictive capacity of these pre-existing pathological conditions was less good than neopterin: hypertension (Specificity=77.6%; Sensitivity=64.1%), diabetes (Specificity=43.4%; Sensitivity=82.6%), cardiovascular disease (Specificity= 19.7%; Sensitivity=92.3%).

Neopterin also acted independently of hospital wards at patients’ admission as shown in **Supplementary Figure S1A**, suggesting that neopterin level was not associated with the initial clinically determined disease status. However, during the infection period, disease severity can be assessed according to disease duration and length of hospital stay. Both parameters were slightly associated with serum neopterin concentration, suggesting that neopterin could help the stratification of patients at higher risk of pernicious outcome (**Supplementary Figures S1B, C**).

Therefore, to evaluate patients’ clinical course, we estimate the predictive potential of elevated neopterin levels. According to the level of neopterin measured in each patient at their arrival to hospital, the probability of survival was significantly reduced for individuals with high systemic neopterin levels (**Figure 2D**; AUC=0.94). The test has 64% specificity and 100% sensitivity with a cut-off value set at 53 nmol/L.

**Figure 3:**
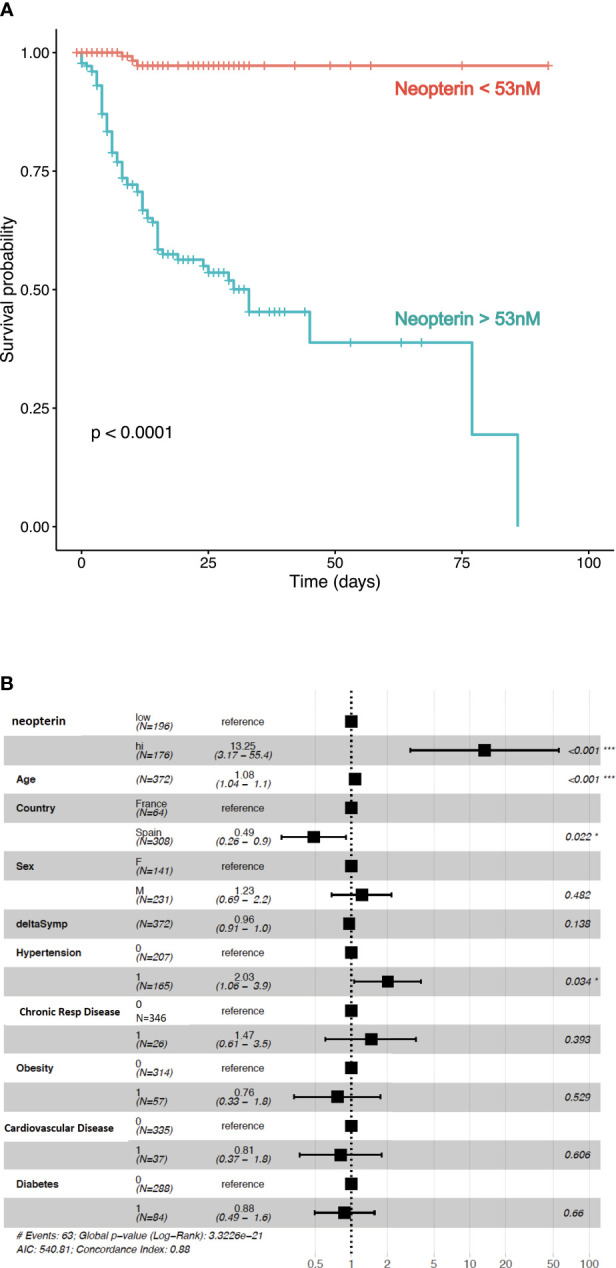
Neopterin is a biomarker of fatal outcome in SARS-CoV-2 infected patients. **(A)** Time from symptoms onset to discharge (or death) was examined in this multi-centric study. Survival curves for Covid-19 patients with high (>53nm) or low (<53nM) serum concentration of neopterin at hospital admission. Kaplan-Meier estimator graph with log-rank based statistics. **(B)** Cox proportional hazard model was used to analyze the effect of confounders, such as age, sex, country, diabetes, obesity, hypertension, respiratory disease and cardiovascular disease. Proportional hazard ratios are indicated along with their 95% confidence intervals.

Based on 53nM as threshold value, Kaplan-Meier survival curves were different between patients with low and high levels of systemic neopterin (**Figure 3A**; p<0.0001). We therefore wanted to evaluate if other confounding variables could impact on the survival analysis. We conducted a Cox proportional hazard model, which beyond neopterin, included age, gender, country, time since initiation of symptoms, hypertension, chronic respiratory disease, obesity, cardiovascular disease and diabetes. Of note, these are all variables readily assessable on the day of hospitalization. SARS-CoV-2 patients with systemic neopterin levels higher than 53nM had 13-fold higher risk of death (hazard ratio (HR), 13.25; 95%CI, 3.17-55.4; p<0.001) when compared with patients with systemic neopterin levels below 53nM (**Figure 3B**). Country, hypertension and in particular age appear to be risk-factors of non-survival (HR 0.49, 2.03 and 1.04; p=0.02, 0.03 and <0.001, respectively). We then assessed if those variables were correlated with neopterin levels. As shown, neopterin was not associated with neither country (**Figure 2C**) nor age and hypertension status (**Supplementary Figure S2**). Of note, although high neopterin was found primarily in old age and individuals suffering from hypertension, the inverse was not true. Indeed, many individuals of old age or suffering from hypertension did not display high levels of neopterin and nor did they die from SARS-CoV-2 infection.

## Discussion

Evidence to date is that approximately 80% of individuals with Covid-19 have a mild disease, i.e. a respiratory tract infection with or without pneumonia, and most of them recovered. In 14% of cases, Covid-19 develops into a more severe disease requiring hospitalization while the remaining 8% of cases experience critical illness requiring intensive care. The mortality of patients hospitalized due to Covid-19 in Europe is approximately 4% and more than 10% in the oldest population (source: ECDC). Monitoring and estimating resource-needs is crucial to ensure that healthcare systems have the capacity to respond to acute surges in cases. Today, clinical decision-making during Covid-19 disease is mainly based on medical parameters since no biomarkers can be used to evaluate the risk for respiratory distress or to decide on adapted care for vulnerable patients.

Here, we demonstrate that neopterin can serve as a predictive biomarker of mortality post SARS-CoV-2 infection, enabling discrimination between patients who will recover and those at risk of fatal clinical outcome. Of note, neopterin level was measured at arrival to hospital before any treatment could interfere. The fact that the study was conducted on two independent cohorts from Spain (n=309) and France (n=65), which could imply different initial health status and medical follow-up, makes us confident that neopterin is a reliable prognostic biomarker for stratifying SARS-CoV-2 infected patients according to disease severity. Indeed, it has been reported that serum neopterin levels measured in patients suffering from mild disease was lower than the concentration found in severe patients (Robertson et al., 2020; Ozger et al., 2021). Another recent study based on 115 patients from Austria showed that SARS-CoV-2-infected patients exhibiting high level of systemic neopterin (>45nM) were more prone to require mechanical ventilation and were at higher risk of intensive care unit admission (Bellmann-Weiler et al., 2021).

Neopterin measurement at hospital admission, identifying patients at risk of a severe disease course (high neopterin levels, >53nM), can serve as an urgently required tool to guide the triage of infected patients and ensure that critical care beds and specialized hospital wards are dedicated to their treatment (Hailemichael et al., 2021). In doing so, healthcare resources will be put to best use during pandemic peak periods and the pressure on specialized hospital wards will be reduced. Infected patients with low levels (<53nM) of neopterin would be considered at low risk and can return to home quarantine and remote follow-up. With a 53nM cut-off value neopterin is able to predict all fatal clinical outcomes (100% sensitivity), but at this threshold value the specificity is 64%. Therefore, a number of false positives are to be expected, which makes it relevant to consider a third stratification level representing patients with intermediate levels of neopterin who should be kept under perpetual observation in a classic hospital wards to assure their follow-up.

Neopterin is induced through IFN-γ signaling. Its concentration might be considered an indicator of systemic immune activation of both lymphoid (T and NK cells) and myeloid cells (monocytes and macrophages). Moreover, neopterin can also indicate oxidative stress (Murr et al., 1999) since its synthesis has been shown to correlate with reactive oxygen species (ROS) production (Nathan, 1986). IFN-γ is an initiator of pro-inflammatory immunity. It is a primary inducer of TNF-α expression (Vila-del Sol et al., 2008). TNF−α is initially produced as surface bound precursor of TNF-α. Mature TNF-α is released through the proteolytic activity of TNF-α Converting Enzyme (TACE), also called ADAM-17. ADAM17 is a sheddase involved in a range of biological phenomena, which is tightly regulated through G-protein coupled receptor (GPCR) activity (Lorenzen et al., 2016). A number of ligands may induce this activity, including complement, nucleotides and hyperoxia (Oarhe et al., 2015). Once cleaved by ADAM17, mature TNF-α may induce signaling leading to activation of the NF-κβ pathway and inflammation. Interestingly, both IFN-γ and TNF-α induce the enzyme guanosine triphosphate cyclohydrolase I (GTPCH) which catalyze the transformation of guanosine triphosphate (GTP) to neopterin (Eisenhut, 2013). Neopterin may therefore serve both as a biomarker of an early inflammatory state based on IFN-γ signaling, which should be associated with a large quantity of surface bound TNF-α ready for secretion upon an acute event, such as viral infection and ADAM17 induction. This therefore suggests that high neopterin levels may be associated with a high risk of developing an intense inflammatory reaction, such as the cytokine storm observed in the late phase of severe SARS-CoV-2 infection (Hailemichael et al., 2021). Distinct cytokine profiles have been described to be associated with COVID-19 severity and differentially predictive of mortality according to oxygen support modalities (Dorgham et al., 2021). Based on cytokine profiling performed on our French patients, we observed multiple positive correlations between cytokines and neopterin which revealed 4 clusters. Neopterin was member of a cluster also including IFN-γ, IL-22, IL-18 and GM-CSF (**Supplementary Figure S3**). This may suggest important innate pathways which could pinpoint physio-pathology mechanisms involved in Covid-19.

The expectation is that adjusting the level of care to the resilience status of infected patients facing Covid-19 will accelerate functional recovery, reducing the time spent at hospitals and the frequency of re-hospitalization, therefore having socio-economic benefits. Measurement of neopterin concentration in various body fluids is easily applicable to the standards of routine laboratories, especially since this fast procedure does not require high amount of biological material, nor specific high-cost equipment. This warrants the accessibility of this biomarker assessment to a large number of potential patients. Here, we measured neopterin in patients’ serum which results in an accurate estimation of disease and hence prognosis; however, this molecule is biologically and chemically stable in all body fluids; future studies should therefore assess if non-invasive salivatory or urinary measurements could also constitute a reliable assay to predict clinical outcome in this context for rapid testing. Preliminary results obtained from 10 patients did not show high level of neopterin in broncho-alveolar lavages (**Supplementary Figure S4**). A recent study showed that the increased fecal neopterin parallels gastro-intestinal symptoms in Covid-19 (Grabherr et al., 2021). In Covid-19 patients with neurologic symptoms, an unusual pattern of marked central nervous system inflammation was observed in association with increased cerebrospinal fluid neopterin levels (Edén et al., 2021).

In conclusion, we propose that neopterin should be implemented as a rapid and easy clinical test to guide the allocation of health care resources during pandemics and aid in adapting the treatment for patients with an increased risk of adverse medical outcomes. It is presently unknown if neopterin and associated hyper-inflammation is a surrogate immune correlate or if it has causal impact on disease progression. Future studies should examine high-risk patients, who may gain most from more specialized medical care such as anti-inflammatory therapy.

## Data Availability Statement

The raw data supporting the conclusions of this article will be made available by the authors, without undue reservation.

## Ethics Statement

The studies involving human participants were reviewed and approved by local ethical committee of Sorbonne-Université/Assistance Publique des Hôpitaux de Paris for standard hospitalized patients (n° CER2020-21) and ICU patients (n° CER2020-31). Similarly, ethical approval was obtained in Spain by local ethical committee (n°PI20-20). The patients/participants provided their written informed consent to participate in this study.

## Author Contributions

MC and KD performed experiments. ML designed research, analyzed the data and wrote the paper. PQ, AM, LR, HV, AG, BQ and JBa collected and analyzed clinical data. EM-C, AM, ZA, FA, JM, HV, JBo, and C-EL recruited patients GG, AM, AG, FA, and EM-C designed clinical protocol and ethical assessment. DS, AG, BC, CC, and GG provided financial support. DS designed research, performed experiments, analyzed the data and wrote the paper. All authors contributed to the article and approved the submitted version.

## Funding

This work was supported by the SARS-CoV-2 Program of the Faculty of Medicine from Sorbonne University (grant i-Covid), by Fondation de France “Tous unis contre le virus”, framework Alliance (Fondation de France, AP-HP, Institut Pasteur) in collaboration with Agence Nationale de la Recherche (ANR-Flash-Covid19).

## Conflict of Interest

ML and DS are inventors of patent EP20305794.8.

The remaining authors declare that the research was conducted in the absence of any commercial or financial relationships that could be construed as a potential conflict of interest.

## Publisher’s Note

All claims expressed in this article are solely those of the authors and do not necessarily represent those of their affiliated organizations, or those of the publisher, the editors and the reviewers. Any product that may be evaluated in this article, or claim that may be made by its manufacturer, is not guaranteed or endorsed by the publisher.
