# Supplementary material for: Elevated Neopterin Levels Predict Fatal Outcome in SARS-CoV-2-Infected Patients
Source: Front Cell Infect Microbiol. 2021 Aug 23;11:709893. doi: 10.3389/fcimb.2021.709893 (PMC8419218; doi:10.3389/fcimb.2021.709893)
Supplement: Supplementary file 1 [file DataSheet_1.pdf]

Supplementary data

Elevated neopterin levels predict fatal outcome in SARS-CoV-2-infected patients

Manon Chauvin[1*], Martin Larsen[1*], Bibiana Quirant[2,3], Paul Quentric[1], Karim Dorgham[1], Luca Royer[1], Hélène Vallet[1,4], Amelie Guihot[1,5], Béhazine Combadière[1], Christophe Combadière[1], Jaume Barallat[6], Julien Mayaux[7], Charles-Edouard Luyt[8], Alexis, Mathian[1,9], Zahir Amoura[1,9], Jacques Boddaert[1,10], Fernando Armestar[11,12], Guy Gorochov[1,5], Eva Martinez-Caceres[2,3], Delphine Sauce[1#]

Affiliations:

[1] Sorbonne Université, Inserm, Centre d'Immunologie et des Maladies Infectieuses, Cimi-Paris, F-75013, Paris, France.

[2] Division of Immunology, Germans Trias i Pujol University Hospital and Research Institute, Campus Can Ruti, Badalona, Spain.

[3] Department of Cellular Biology, Physiology and Immunology, Universitat Autònoma de Barcelona, 08193 Bellaterra (Cerdanyola del Vallès), Spain.

[4] Assistance Publique-Hôpitaux de Paris (AP-HP), Hôpital Saint-Antoine, Unité de Gériatrie Aigue, F-75012, Paris, France.

[5] Assistance Publique-Hôpitaux de Paris (AP-HP), Groupement Hospitalier Pitié-Salpêtrière, Département d'Immunologie, F-75013, Paris, France.

[6] Biochemistry Department, Germans Trias i Pujol University Hospital, Badalona, Spain.

[7] Assistance Publique-Hôpitaux de Paris (AP-HP), Groupement Hospitalier Pitié-Salpêtrière, Service de Médecine Intensive–Réanimation et Pneumologie, F-75013, Paris, France.

[8] Service de Médecine Intensive Réanimation, Institut de Cardiologie, Assistance Publique–Hôpitaux de Paris (APHP), Sorbonne-Université, Hôpital Pitié–Salpêtrière, Paris, France.

[9] Service de Médecine Interne 2, Institut E3M, Assistance Publique Hôpitaux de Paris (AP-HP), Hôpital Pitié-Salpêtrière, F-75013 Paris, France.

[10] c, Assistance Publique–Hôpitaux de Paris (APHP), Sorbonne-Université, Hôpital Pitié–Salpêtrière, Paris, France.

[11] Critical Care Department, Germans Trias i Pujol University Hospital, Badalona, Spain.

[12] Department of Medicine Universitat Autònoma de Barcelona, 08193 Bellaterra (Cerdanyola del Vallès), Spain.

* Equally contributed to the work

Supplementary Table S1: Patients' description

Group	CTRL (n=256)	COVID-19 (n=374)	p
**Age**			
Mean (SD)	63 (13)	61 (15)	0.076
**Gender**			
Female	44% (113)	38% (142)	0.13
**Service**			
Hospital	n.a	25.7% (96)	
Emergency	n.a	55% (206)	
ICU	n.a	19.3% (72)	
**Past Medical History**			
Cardiovascular Disease	n.a	10.1% (38)	
Hypertension	n.a	44.4% (166)	
Diabetes	n.a	22.7% (85)	
Obesity	n.a	15.2% (57)	
Chronic Respiratory Disease	n.a	7% (26)	
**Final Clinical Outcome (%)**			
Discharge	100	80% (298)	
Death	0	20% (76)	
Median Length of Stay (days)	n.a	15	
**Neopterin**			
Mean (SD)	9.5 (3.8)	56 (38)	<0.001

A.

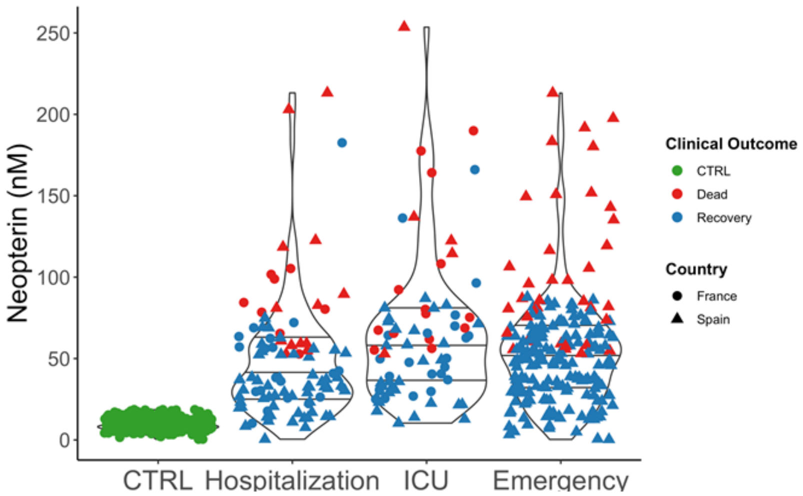
Neopterin (nM)
250
200
150
100
50
0
CTRL
Hospitalization
ICU
Emergency
Clinical Outcome
CTRL
Dead
Recovery
Country
France
Spain

B.

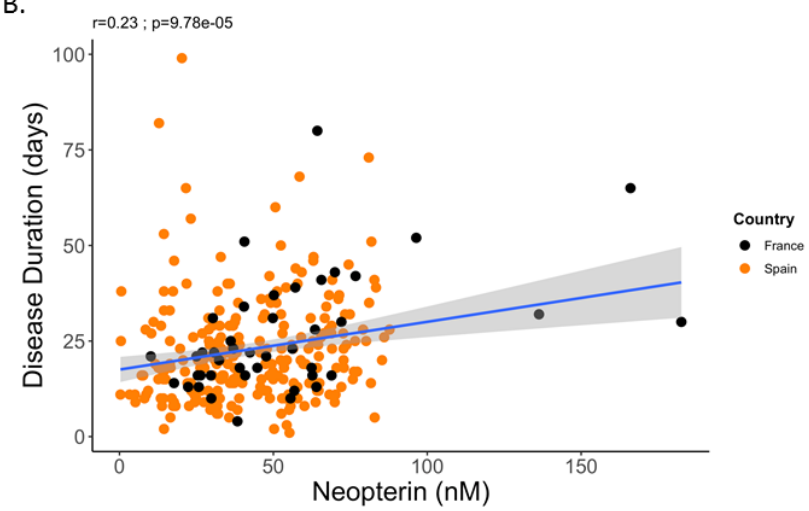
r=0.23 ; p=9.78e-05
Disease Duration (days)
100
75
50
25
0
0
50
100
150
Neopterin (nM)
Country
France
Spain

C.

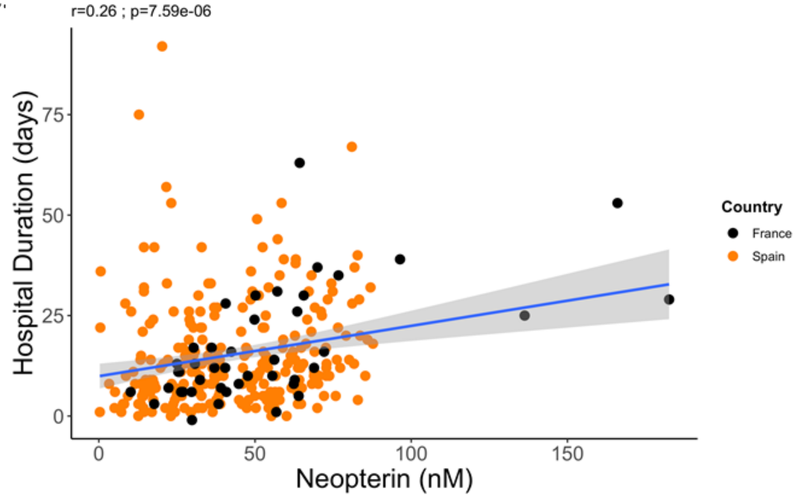
r=0.26 ; p=7.59e-06
Hospital Duration (days)
75
50
25
0
0
50
100
150
Neopterin (nM)
Country
France
Spain

Supplementary Figure S1: Neopterin is associated with disease severity

A. Violin plots representing serum neopterin levels (in nM) stratified according to hospital wards at admission for French (circles; n=65) or Spanish (triangles; n=309) SARS-CoV-2 infected patients. Final outcome is represented by colored symbols (blue as recovery; red as non-survivors). Green symbols represent the 256 healthy volunteers.

B. Scatter plot representing the correlation between the level of neopterin measured at day 0 (nmol/L) and the disease duration (days). r=0.23; p=0.0001.

C. Scatter plot representing the correlation between the level of neopterin measured at day 0 (nmol/L) and the length of hospital stay (days). r=0.26; p<0.0001.

French and Spanish patients are represented with circles and triangles, respectively.

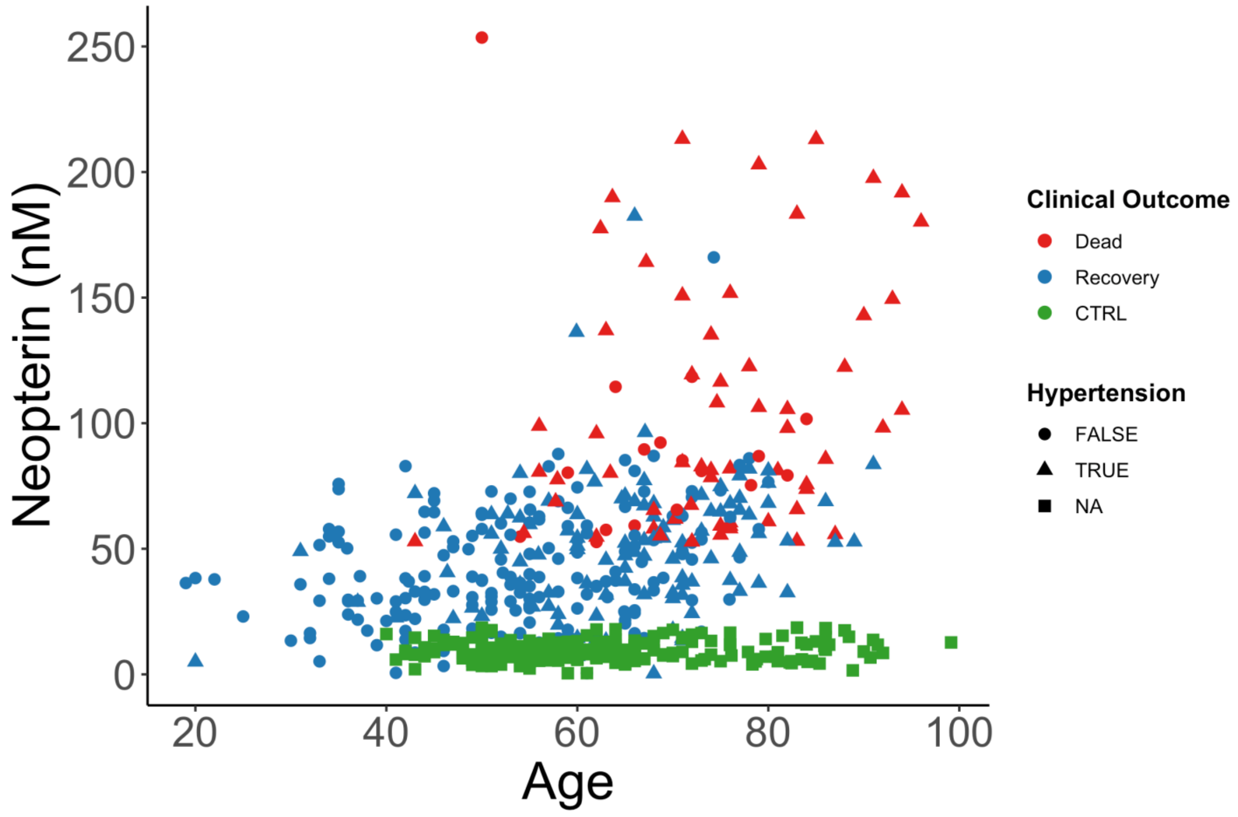

Supplementary Figure S2: Neopterin is not associated with age nor hypertension

Scatter plot associating serum neopterin levels (in nM) and age. Final outcome is represented by colored symbols (blue as recovery; red as non-survivors). Green symbols represent the 256 healthy volunteers. The shape of the symbols takes into account the hypertension status of the infected patients (no hypertension as circles; hypertension as triangles).

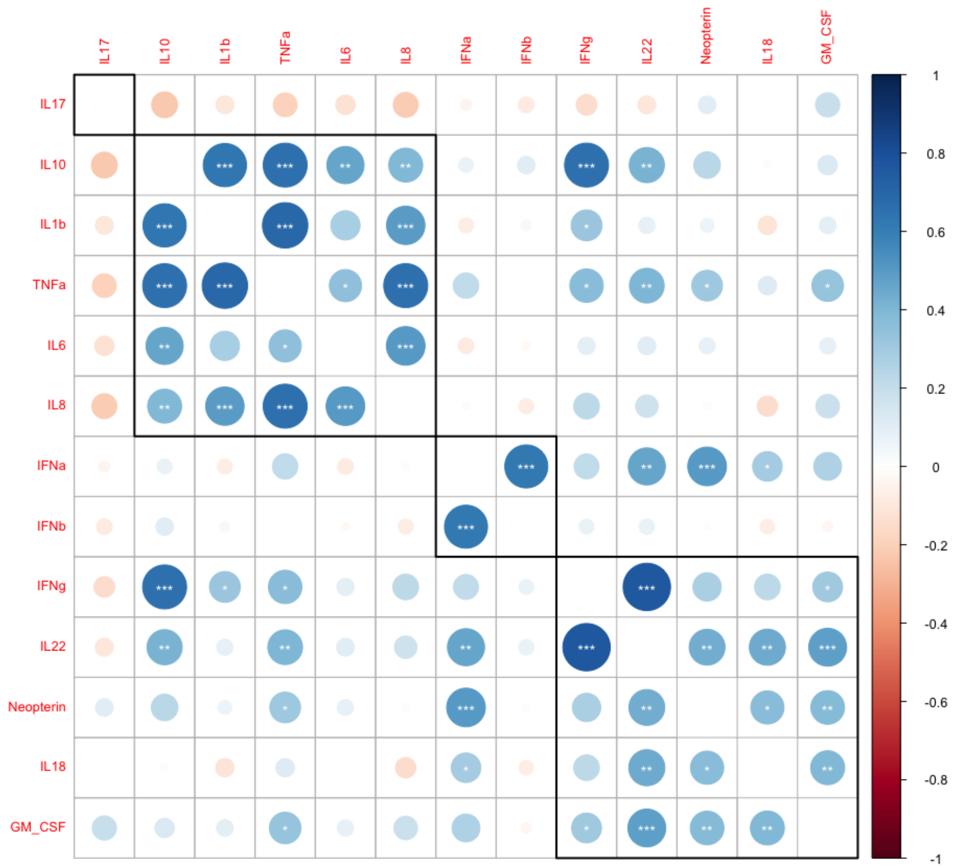

Supplementary Figure S3: Neopterin is associated with cytokine secretion

Spearman correlation plots across 13 markers of inflammation measured in the French samples at hospital admission. The size and color of the dots correspond to the correlation coefficient between each pair of biomarkers. The absence of a dot indicates a non-significant correlation defined as an adjusted p-value > 0.05.

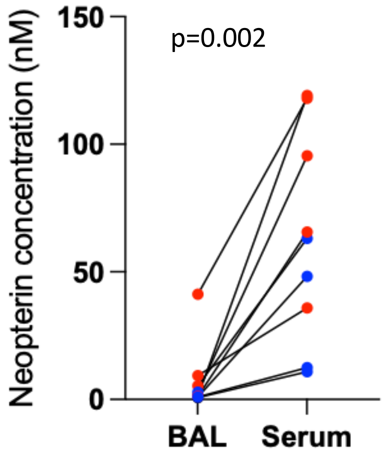

Supplementary Figure S4: Low level of neopterin in broncho-alveolar lavages

Paired scatter plot representing neopterin levels (in nM) measured in both BAL and serum of 10 ICU patients. The p-value is derived from a non-parametric paired Wilcoxon statistic.
